# A feasibility and acceptability study of liberate: an online, peer-supported, psychoeducational intervention for ultra processed food addiction

**DOI:** 10.3389/fpsyt.2025.1620372

**Published:** 2025-10-29

**Authors:** Ellen Bennett, Deborah Lycett, Maxine Whelan, Erin L. Bellamy, Sara Banks, Riya Patel

**Affiliations:** ^1^ Centre for Healthcare and Communities, Coventry University, Coventry, United Kingdom; ^2^ Public Health Collaboration UK, London, United Kingdom; ^3^ School of Psychology, University of East London, London, United Kingdom; ^4^ Diabetes Research Centre, University of Leicester UK, London, United Kingdom

**Keywords:** addiction, food addiction, food addiction abstinence or harm reduction, low carbohydrate, substance use disorder, ultra processed food substance use disorder

## Abstract

**Introduction:**

Ultra-processed food addiction (UPFA) is a growing popular topic in the research arena. There are calls for its classification for diagnosis as a substance use disorder and behavioural disorder. Although evidence for UPFA is increasing, effective interventions remain scarcely available. This is a feasibility ty -50and acceptability study of “Liberate,” an online, peer-supported psychoeducation intervention for adults self-identifying with UPFA.

**Methods:**

A single-group, pre- and post-mixed methods study with 6-month follow-up was used. Participants (n=86) attended a 6-week coach-led online programme which comprised educational sessions, peer-to-peer support, and a personalised abstinence or harm reduction approach to dietary changes. Quantitative data included UPFA symptom measurements (YFAS 2.0, CRAVED), mental wellbeing (WEMWBS), and anthropometrics (weight, kg, and body mass index [BMI]). Acceptability was assessed qualitatively using semi-structured interviews which were then analysed thematically using the Theoretical Framework of Acceptability.

**Results:**

Recruitment and retention rates were acceptable. Statistically significant improvements were observed in UPFA symptoms (YFAS mean reduction: −3.4; 95% CI: −4.5, −2.3), CRAVED scores (−1.4; 95% CI: −1.8, −0.9), and mental wellbeing (5.4; 95% CI: 3.2, 7.6) from baseline, post-intervention, and sustained at 6-month follow-up. BMI and weight change also showed a statistically significant reduction, but this was clinically negligible. Thematic analysis revealed high acceptability, with participants reporting greater self-awareness, reduced impulsive eating and eating behaviours, and increased confidence in managing UPFA symptoms. They found Liberate to be a psychologically safe and non-judgemental space, becoming aware that it was “not my fault,” and developing hope of a future beyond the intervention. Peer support and education on the effects of addiction on the brain were reported as being particularly beneficial. They expressed a desire that healthcare professionals would refer to Liberate.

**Conclusion:**

This study finds “Liberate” an online UPFA intervention, to be a feasible and acceptable intervention. Further investigation through a randomised controlled trial would be needed to establish causality, long-term effectiveness, and potential scalability.

## Introduction

Food addiction, now known as ultra-processed food addiction (UPFA), the term we will refer to in this study, is a behavioural and neurobiological condition in which individuals compulsively consume highly processed foods in a manner similar to substance addiction. This includes cravings, loss of control, tolerance, withdrawal symptoms, and continued use despite negative consequences. Although not formally recognised in diagnostic manuals such as the DSM-5 or ICD-11, it is measurable using the Yale Food Addiction Scale (YFAS). In a non-clinical sample, it has been estimated to affect approximately 14% of adults and 12% of children globally (1–3). In clinical samples, the estimation rises significantly; 55% of those with anorexia, 63% of those with binge eating disorder, 84% of those with bulimia are said to be affected by the symptoms of UPFA, and 30% of those with type two diabetes mellitus are estimated to have symptoms of UPFA (4, 5). UPFA research has been growing over the past six decades, with a notable increase in recent years (6). There is a broad consensus that addictive-like eating exists, driven by biological and behavioural mechanisms as well as industry practices in refining foods to make them hyperpalatable and “addictive”. This is similar to processes seen in substance use disorders (SUDs) (7). Evidence suggests UPFA meets SUD criteria, including eating more than intended due to strong cravings; inability to cut back; and continued use despite harm. Also, individuals may neglect responsibilities and relationships to maintain the addiction and experiencing withdrawal symptoms (such as irritability) when unable to access specific foods. This has been reported many times in narrative and systematic reviews of the literature (8–11), with many researchers calling for its classification as both a behavioural and substance-based addiction (12).

As research has advanced, specific standardised diagnostic tools such as the Yale Food Addiction Scale (YFAS) and CRAVED, an ICD-10 based food behaviour questionnaire, have been developed to assess UPFA and understand its unique characteristics (13–15). Reviews of the studies that have measured UPFA using these scales suggest that the global prevalence of UPF addiction in a non-clinical sample of adults could be between 14% and 20%, comparable with other SUDs (7, 8).

A positive association between UPFA, measured with the YFAS, and UPF intake has been observed in young people (16). A review of more than 50 publications aimed at distinguishing binge eating disorder (BED) from UPFA found that, whilst distinct characteristics exist between the two, for example, depressive symptoms and brooding rumination being more associated with BED, and heightened focus on taste and pleasure in UPFA, there are overlapping features. This highlights the need for further research to clearly differentiate the two conditions. Systematic reviews have found a higher prevalence of UPFA in individuals with BED (17, 18), and a meta-analysis of 272 studies found that UPFA was higher in those with a diagnosis of BED and concurrent weight disorders (19).

Additionally, research on women with eating disorders has shown that UPFA is associated with recurrent binge eating episodes, eating disorder (ED) severity, and lower interoceptive awareness, further indicating an overlap between ED and UPFA (20). Notably, some individuals with UPFA do not have a diagnosed ED. UPFA can manifest as either binge eating or grazing behaviours, making accurate diagnosis challenging (2). It has also been assumed that UPFA is more common in individuals with obesity. However, studies have found a higher prevalence in individuals with Class III obesity, people who are underweight, and people with a current eating disorder, suggesting other factors contribute to UPFA (21, 22).

A formal diagnosis cannot occur without recognition of UPFA as a condition; as a result, there has been little research to date on targeted interventions. There is, however, evidence supporting the appropriateness of existing weight loss interventions for treating UPFA.

For example, for individuals who are obese, bariatric-metabolic surgery is an effective intervention for reducing UPFA symptoms, with a systematic review and meta-analysis reporting notable improvements post-surgery (23). Among those undergoing bariatric-metabolic surgery, UPFA rates dropped from 32% to 15% (22). Bariatric surgery is highly invasive with potential long-term complications, and although the intervention has been shown to reduce UPFA symptoms, the weight regain and reports of old eating behaviours returning suggests that the relief from UPFA symptoms is only temporary (24–26). Post-surgery psychological support is often necessary for these individuals. Psycho-behavioural interventions have also been investigated. A randomised controlled trial (RCT) of using neurological feedback in women living with obesity and UPFA showed that it was more effective at reducing food cravings than the placebo but its long-term effectiveness is unclear (27). Therefore, it is essential to develop targeted interventions that can address psychosocial support needs.

Another widely accessed intervention for UPFA is Overeaters Anonymous (OA). OA is a face-to-face and online self-help group with approximately 54,000 members. OA is structured around a peer-led, 12-step, spirituality-based framework and operates without direct involvement from healthcare professionals such as dietitians and psychologists (28). OA promotes and practices complete abstinence from identified trigger foods associated with uncontrolled consumption (29). Recovery is supported through explicit and implicit tools such as telephone calls among members, OA written resources, and the power of belonging to the group (30–32). Online support groups and psychosocial interventions are helpful (31), but little research has explored the efficacy of dietary and nutritional strategies in the treatment of UPFA. Furthermore, targeted interventions that can address specific psychosocial support needs may be of more benefit but are yet to be investigated.

Dietary interventions, particularly focused on carbohydrate reduction, may provide longer-term relief for UPFA. A feasibility case series showed significant improvements in weight loss, binge eating episodes, and UPFA symptoms (such as food cravings and lack of control) with individuals maintaining their improvements for 17 months (33). An RCT investigating a personalised goal setting telehealth nutrition intervention to improve diet quality in those with more than three UPFA symptoms found that this was more effective than a non-personalised intervention or the control group (34). A pilot study of a very low-calorie ketogenic diet (VLCKD) in women with binge eating and/or UPFA symptoms resulted in weight loss and the absence of UPFA and BED symptoms at study end (35). A qualitative study exploring the ketogenic diet’s effects on mental health outcomes suggests that abstinence from UPFs was key to reducing cravings (36). Additionally, one study has shown a low-carbohydrate diet combined with psychoeducation results in significant improvements in mental well-being for those with UPFA (13).

The studies cited collectively suggest that a low-carbohydrate, ketogenic diet and/or complete abstinence from UPFA, together with psychoeducation delivered in an online forum, may help treat UPFA. As a result, in developing the programme Liberate, we provided opportunity for complete abstinence, in a personalised way, from foods that individuals considered problematic. This focus on abstinence is first and foremost in the Liberate programme. Framing this within psychological education and peer-to-peer support is the back bone of this intervention. Secondly, it is encouraged that the participants follow a whole food diet preferably low-carbohydrate eating, but this is optional since there was no fixed level of carbohydrate individuals had to consume nor was it necessary that they enter a ketosis. This paper reports on a pre- and post-intervention feasibility and acceptability study of Liberate, run by the Public Health Collaboration (UK Charity No. 1171887). It was hypothesised that Liberate would be a feasible and acceptable intervention for those self-identifying with UPFA.

### Aims and objectives

This study aimed to investigate the feasibility and acceptability of “Liberate,” an online psycho-education course for ultra-processed food addiction (UPFA) with peer-to-peer support.

The objectives were to:

- Assess recruitment and retention rates, adherence rates, time required to recruit participants, and percentage of participants identified as “addicted to food”
- Determine the effect size of the change in addictive behaviour, eating disorder scores, mental well-being scores, and anthropometric measures (weight and body mass index [BMI]) to inform a subsequent trial sample size calculation
- Explore participant acceptability of Liberate

## Methods

### Study design

A mixed methods design was used for this single-group pre–post-test feasibility study with nested acceptability component.

### Participant eligibility

#### Inclusion criteria

- Aged ≥18 years
- BMI of >20 kg/m^2^
- Willing and able to provide informed consent to participate and to adhere to the study procedures
- Able to read and speak English
- Access to a computer with camera and microphone

#### Exclusion criteria

- Currently experiencing an active episode of anorexia nervosa, characterised by significant weight loss, ongoing restrictive eating behaviours, and/or medical instability. This presentation requires urgent referral to a specialist eating disorder service for assessment and management.
- Likely to have bariatric surgery in the next 6 months or have had bariatric surgery in the last 12 months as this may skew the outcome of BMI and weight.
- Pregnant or planning to become pregnant in the next 6 months as this will skew the outcome of BMI and weight.

Those who did not meet the criteria to take part in this study were signposted to other food addiction support networks, such as Public Health Collaboration, BEAT, and OA.

#### Sample size

Given the aims and scope of a feasibility trial, a sample size of 135 participants was considered sufficient overall with no formal power calculation required (37). Although a sample size of 135 was originally sought, the study managed to enrol 105 eligible participants within the recruitment window. This was considered sufficient for feasibility purposes, as the original sample size was inflated to allow for attrition.

For the embedded qualitative study, a sample size of 13 participants was considered appropriate using a 10 + 3 criterion until saturation was reached (38).

#### Recruitment

Participants were recruited through posts from the service provider (Public Health Collaboration) on social media platforms including Instagram, X (formerly Twitter), and TikTok.

Potential participants were invited to attend a live, online information session (lasting 30 minutes, hosted on Microsoft Teams) in January 2024. These information sessions (eight delivered in total over 6 weeks) were held at the same time slots as the intervention would happen to ensure accessibility. After the information session, if potential participants remained interested in taking part, individuals were signposted to the study information sheet, consent forms, and the screening questionnaire to determine their eligibility.

### Intervention

Full details of the Liberate intervention, using the TiDieR checklist (39), session content, and the behaviour change techniques (40) used, are provided in the **Supplementary Material**.

Briefly, Liberate (developed by Ellen Bennett RD) is a community-based intervention for people living with UPFA delivered by a community charity organisation (Public Health Collaboration; Charity No. 1171887). Liberate uses online, video conferencing software to deliver an eight-session (over 6 weeks), coach-led, educational programme with 1 year of peer-to-peer support. Liberate provides education around the scientific mechanisms underpinning brain activity in addiction, in lay terms, providing the user with more scientific and non-judgemental explanations for their addiction, enabling them to have a deeper understanding of UPFA and learn about strategies to address UPFA including psychological techniques, peer-to-peer social support, and dietary changes. Liberate encourages a personalised abstinence approach, using a traffic light system with red foods being problematic and not being able to eat “sanely” and are typically but not always, and ultra processed foods high in carbohydrates and/or fats such as cakes, biscuits, sweets, pastries, breads, pizza, etc., and orange being that the individual can tend to overeat on them but are not as problematic as red foods. These foods typically are minimally processed such as salted nuts and cheese. The amber foods need some boundaries in place to ensure they are not routinely eating them over a varied diet and in manageable quantities; green foods are foods that can be consumed without difficulties. These typically are whole foods without added high amounts of fats carbohydrates and salt. However, this is individual to the participant. This tool supported complete abstinence from problem foods where appropriate, while also offering a harm reduction approach as an alternative. From a dietary point of view, it is more important for the individual to recognise their own addictive foods and replace them with minimally processed foods as part of a personalised dietary strategy. Abstinence is considered preferable by many with UPFA because it mirrors approaches used in substance addiction treatment, where complete removal of the addictive agent often leads to better control, fewer relapses, and reduced psychological distress compared with moderation. Participants could also choose to follow a low-carbohydrate food plan, but there was no specified amount and no mandatory requirement to enter ketosis.

Each session lasted 90 min, beginning with participants having the opportunity to share thoughts and feelings with the group about how the previous week had gone and any difficulties or successes with food. After this, the facilitator presented the educational element. This was displayed on the screen, and the facilitator discussed each topic (e.g., the non-addicted brain vs. the brain in active addiction, the effect of cortisol and insulin in the brain and how that contributes to addiction symptoms). Participants were encouraged to listen and ask questions. Before sessions ended, there were opportunities for questions followed by 3 min of self-reflection “quiet time”. Participants were encouraged to set personal goals, engage in “homework” between sessions, consisting of written reflection tasks, peer contact, and videos to watch to enhance their learning.

### Data collection

#### Participant characteristics

Data were collected on sex, age, ethnicity, education level, employment status, body mass index (BMI), and prior history of addictive eating.

### Primary outcomes

#### Feasibility

Feasibility was assessed by collecting data on recruitment and retention rates, adherence rates, time required to recruit participants, and percentage of those exposed to the intervention identified as “addicted to food”.

#### Acceptability

Acceptability was evaluated qualitatively using the Theoretical Framework of Acceptability (41). The framework measures acceptability across seven domains: burden, ethicality, intervention, coherence, opportunity costs, perceived effectiveness, and self-efficacy. The Theoretical Framework of Acceptability covers prospective acceptability, concurrent acceptability, and retrospective acceptability (41). Additional questions related to intervention context were added to capture participant understanding and enactment of the intervention. Acceptability interviews lasted no longer than 1 h, were semi-structured, recorded, and took place via video call within 2 weeks of the intervention ending. In addition, participants who dropped out were invited to participate in an interview to explore reasons for dropout.

Field notes were taken by the intervention deliverer and triangulated with the qualitative data to provide insights into the intervention content, setup, and delivery of the intervention.

### Secondary outcomes

Quantitative data were collected and analysed on UPFA behaviours measured using the Yale Food Addiction Scale 2.0 (YFAS) (15), CRAVED (13), and the Binge Eating Scale (BES) (42). Anthropometric measures were weight (kg), height (cm), and BMI (kg/m^2^). Mental Wellbeing was measured using the Warwick and Edinburgh Mental Wellbeing Scale (WEMWBS) (43).

### Quantitative data analysis

Descriptive statistics of recruitment and retention rates, adherence rates, time required to recruit service users, and post support group attendance were carried out.

Mean change with 95% confidence intervals between baseline, end of intervention, and at 6-month follow-up is presented for weight, BMI, YFAS, CRAVED, and WEMWBS. Intention to treat (ITT) analysis was used, with missing data input using baseline observation carried forward.

Change in percentage frequency of participants across the clinical categories of each of the above variables is also presented descriptively.

Repeated measures ANOVA tests were used to test whether the change in these variables were statistically significant. A Bonferroni correction was applied for multiple testing.

These data were analysed using the statistical software package IBM SPSS Statistics for Windows, Version 26.0 (44).

### Qualitative data analysis

Data from the interviews were transcribed verbatim. Data were analysed using reflective thematic analysis (45) whereby transcripts were read and re-read, meanings were coded, and codes were developed into sub-themes and themes. Coding and theme development was mapped to the Theoretical Framework of Acceptability, and open coding was also used.

## Results

### Recruitment

**Figure 1:**
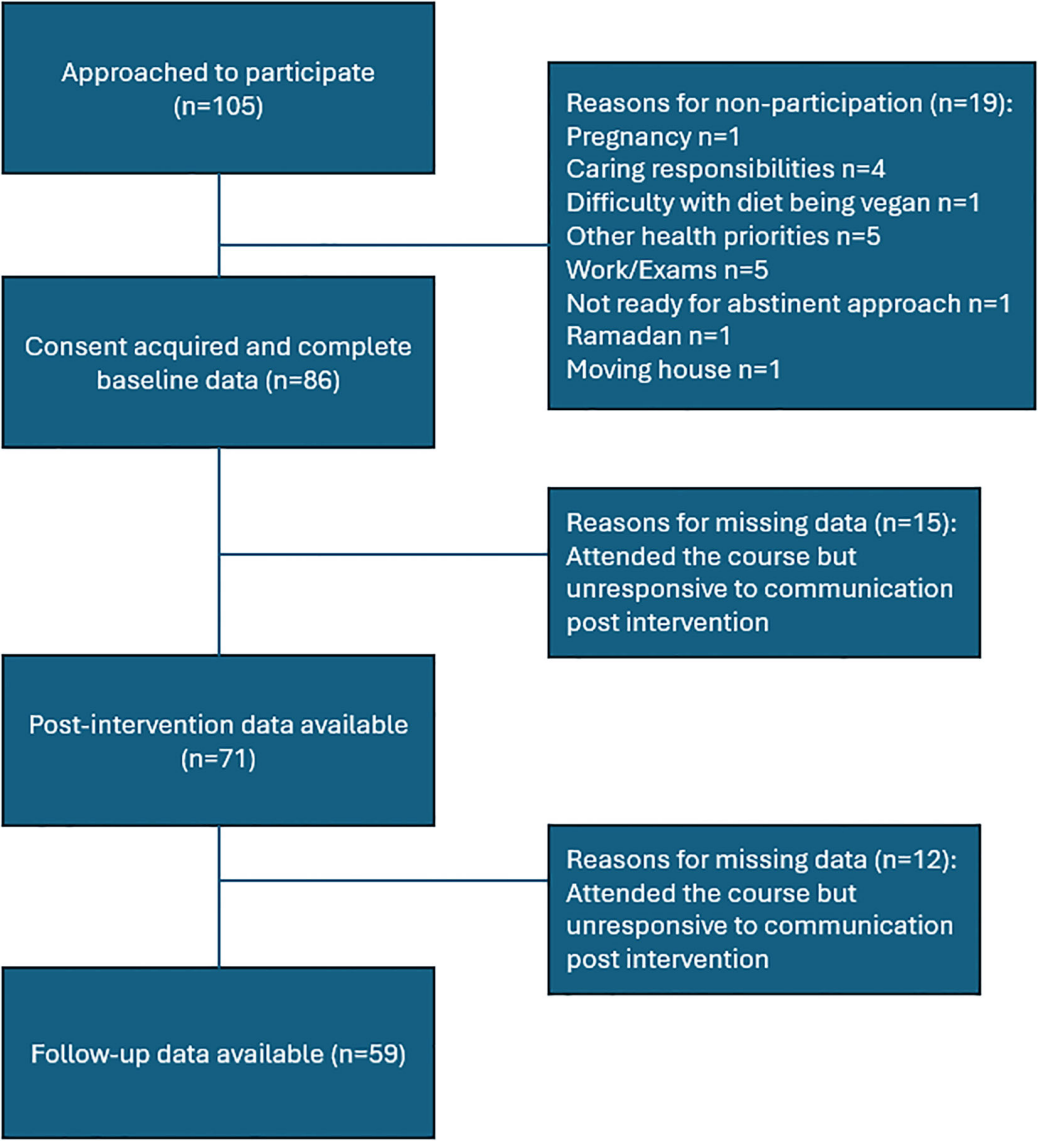
Flow chart of participants.

A total of 105 individuals completed recruitment forms and were qualified for the study. Of those, 86 started the course and 68 attended four or more of the six educational sessions. We were able to collect data on 71 (of 86) participants at the end of the intervention and 59 (of 86) at the 6-month follow-up (**Figure 1**). 

### Participant characteristics

Most of the participants were women (n=79; 91.9%). The two most common age brackets were 35-44 years (n=27; 31.4%) and 45-55 years (n=19; 22.1%). Most of the sample was White British or White Other (n=75; 87.2%). Most participants were educated to undergraduate level (n=26; 30.2%) or postgraduate level (n=38; 44.2%) or were employed (n=60; 69.7%). The majority of participants were in the overweight (n=22; 26%) or obesity class I bracket (n=20; 23%) for BMI. Lastly, one-third of participants had previously heard of or received support for UPFA (n=28; 32.6%) (**Table 1**).

**Table 1:** Baseline characteristics of participants at each data collection point in the study.

	Baseline, n= 86 (%)	Post- intervention data, n=71 (%)	6-month follow-up, n=59 (%)
Sex (%)			
Female	79 (91.9)	65 (91.5)	56 (94.9)
Male	7 (8.1)	6 (8.5)	3 (5.1)
Baseline age (years)			
18-24	1 (1.2)	1 (1.4)	1 (1.7)
25-34	16 (18.6)	13 (18.3)	11 (18.6)
35-44	27 (31.4)	18 (25.4)	14 (23.8)
45-54	19 (22.1)	18 (25.4)	16 (27.1)
55-64	16 (18.6)	14 (19.7)	12 (20.3)
64-75	7 (8.1)	7 (9.9)	5 (8.5)
Ethnicity (%)			
White British or White other	75 (87.2)	61 (85.9)	52 (88.1)
Other	11 (12.8)	10 (14.1)	7 (11.9)
Education (%)			
GCSE	13 (15.1)	13 (18.3)	13 (22)
A-Levels	9 (10.5)	6 (8.5)	4 (6.8)
Undergraduate	26 (30.2)	20 (28.2)	15 (25.4)
Postgraduate	38 (44.2)	32 (45.1)	27 (45.7)
Employment status (%)			
Unable to work	8 (9.2)	7 (9.9)	7 (11.8)
Employed	60 (69.7)	47 (66.2)	39 (66.1)
Self-employed	6 (7)	5 (7.0)	4 (6.8)
Retired	12 (14)	12 (16.9)	9 (15.3)
B-BMI (kg/m^2^)			
Healthy weight	16 (18.6)	12 (16.9)	10 (16.9)
Overweight	22 (26)	19 (26.8)	17 (28.8)
Obesity class I	20 (23)	15 (21.1)	14 (23.7)
Obesity class II	14 (16)	13 (18.3)	9 (15.3)
Obesity class III	14 (16)	12 (16.9)	9 (15.3)
Heard of or had help for addictive eating before (%)			
Yes	28 (32.6)	24 (33.8)	20 (33.9)
No	58 (67.4)	47 (66.2)	39 (67.4)

There were 15 participants who dropped out at the end of the intervention, and a further 12 participants dropped out at follow-up. The baseline characteristics of those who completed the intervention were similar to those who started the intervention apart from age, where there was a reduction in those in the age bracket 34-45 years (31.4% at baseline to 23.8% at the 6-month follow-up) (**Table 1**).

## Quantitative results

### Outcome scores: mean change

#### Yale Food Addiction Score (mean)

There was a statistically significant reduction in food addiction symptoms from baseline to post-intervention as measured by YFAS: mean difference −3.9, (95% CI: −5.0, −2.7). At the 6-month follow-up, there was a slight increase compared with post-intervention: 0.4 (95% CI: −0.5, 1.4). The difference from baseline to 6-month follow-up suggests that the intervention effects were sustained: −3.4 (95% CI: −4.5; −2.3) (**Table 2**).

**Table 2:** Outcome scores and mean changes.

Outcome measure	Baseline n=86	End of intervention=86 ITT	Difference between baseline and end of intervention (mean, 95% CI)	End of follow-up n=86 ITT	Difference between end of follow-up and end of intervention (mean, 95% CI)	Difference between follow-up and baseline (mean, 95% CI)
Primary outcome YFAS score	7.8	4.0	Mean: −3.9 95% CI: −5.0, −2.7*	4.4	Mean: 0.4 95% CI: −0.5; 1.4	Mean: −3.4 95% CI: −4.5; −2.3*
CRAVED score	5.1	3.4	Mean: −1.7 95% CI: −2.2, −1.2*	3.7	Mean: 0.3 95% CI: −0.1, 0.8	Mean: −1.4 95% CI: −1.8; −0.9*
WEMWBs total score	41.9	48.2	Mean: 6.3 95% CI: 8.5, 4.1*	47.4	Mean: −0.9 95% CI: −3.0, 4.1	Mean: 5.4 95% CI: 3.2, 7.6*
BMI kg/m^2^	32.7	32.2	Mean: −0.5 95% CI: −0.9, −0.1*	32.0	Mean: −0.1 95% CI: −0.1; −0.3*	Mean: −0.6 95% CI: −0.9; −0.4*
Weight kg	90.3	89.1	Mean: −1.2 95% CI: −2.1, −0.3*	88.0	Mean: −0.9 95% CI: −2.2, −0.3*	Mean: −2.0 95% CI: −4.0, −0.7*

*P ≤ 0.05.

#### CRAVED score (mean)

There was a statistically significant change in CRAVED score from baseline to post-intervention: mean difference −1.7 (95% CI −2.2, −1.2). There was no significant change from post-intervention to 6-month follow-up, meaning the change was sustained overall, with baseline to 6-month follow-up mean change statistically significant: −1.4 (95% CI −1.8, −0.9).

#### Warwick and Edinburgh Mental Wellbeing score (mean)

In the WEMWBS scores, we saw a statistically significant increase of 6.3 (95% CI: 8.5, 4.1) between baseline and post-intervention, a small decrease between post-intervention and 6-month follow-up (−0.9, 95% CI: −3.0, −4.1). An overall sustained improvement from baseline to 6-month follow-up of 5.4 (95% CI: 3.2, 7.6) was observed. Given that a 3-point increase in WEMWBS is the amount needed to show a clinically meaningful increase in wellbeing a community sample, achieving a 6-point increase at post-intervention and almost having that figure sustained at 6-month follow-up demonstrates that the intervention significantly improved mental wellbeing in a meaningful way.

BMI was reduced from 32.7 to 32.0 kg/m^2^ from baseline to end of intervention: −0.5 (95% CI: −0.8, −0.1). There was another slight decrease from post-intervention to 6-month follow-up falling to a mean of 32.0 kg/m^2^ (−0.1, 95% CI: −0.3, −0.1) and showing an overall reduction from baseline to 6-month follow-up of −0.6 (95% CI: −0.9, −0.4).

Weight fell from a mean of 90.3 to 89.1 kg (−1.2, 95% CI: −2.1, −0.3) from baseline to end of intervention and to 88.0 kg (−0.9, 95% CI −2.2, −0.3) at the 6-month follow-up. Overall weight loss from baseline to 6-month follow-up was significant statistically (−2.0, 95% CI: −4.0, −0.7). However, the clinical impact of 2.0kg over 6 months is questionable.

### Outcome measures: change in clinical categories

**Table 3** shows the data on changes in the clinical classifications of BMI, YFAS, CRAVED, and WEMWBS over the three time points (baseline, post-intervention, and 6-month follow-up). Of particular note, 70% of participants at baseline were scored as having severe food addiction through the YFAS questionnaire. This dropped to 34% at the 6-month follow-up resulting in a 36% reduction overall. Additionally, at baseline, 22% of participants were classified as not having UPFA and by the 6-month follow-up 59% of participants were categorised as not having UPFA. Regarding the CRAVED category, we found a reduction in those who met the screening criteria for UPFA, which decreased from 95% at baseline to 72% at the 6-month follow-up. Those categorised with low mental wellbeing as measured by WEMWBS decreased from 49% at baseline to 30% at the 6-month follow-up, and those in the category for high mental wellbeing increased from 2% at baseline to 12% at the 6-month follow-up.

**Table 3:** Outcome measures: change in clinical categories table.

	Baseline n= (%)		Post-intervention ITT (%)		6-month follow-up ITT (%)	
BMI classes						
Normal weight	16	19%	16	19%	16	19%
Overweight	22	26%	18	21%	25	29%
Obese I	20	23%	27	31%	19	22%
Obese II	14	16%	12	14%	12	14%
Obese III	14	16%	13	15%	14	16%
	86	100%	86	100%	86	100%
YFAS						
NoFA	19	22%	59	69%	51	59%
Mild	5	6%	2	2%	1	1%
Moderate	2	2%	3	3%	5	6%
Severe	60	70%	22	26%	29	34%
	86	100%	86	100%	86	100%
CRAVED						
≥3 yes	82	95%	55	64%	62	72%
<3 yes	4	5%	31	36%	24	28%
	86	100%	86	100%	86	100%
WEMWBS						
Low	42	49%	22	26%	26	30%
Moderate	42	49%	56	65%	50	58%
High	2	2%	8	9%	10	12%
	86	100%	86	100%	86	100%

## Qualitative results

A total of 13 participants’ interview transcripts were analysed; they were predominantly women, heterosexual, and White British n=12 in those categories. The ages ranged from 18 to 64 years. Seven were married, and the remaining six were either never married or widowed. Religious or spiritual beliefs of the interviewees were n=5 Christian, n=1 Jewish, n=3 spiritual, and n=4 reporting no religion. Education status varied among the interviewees n=2 at the GCSE level, n=1 at the A-level, n=5 at the undergraduate level, and the remaining at the post-graduate level n=5. Employment status was n=9 employed and the remaining four not currently in employment or retired. BMI categories of the interviewees also varied with five being in the pre-obesity category, three in the obesity class I and the remaining five in the obesity class III.

Participants’ responses were deductively coded into the domains of the Theoretical Framework of Acceptability (TFA). Experiences of the Liberate intervention were coded and built into subthemes, with some overlap between domains. These findings are described in the text below and evidenced with supporting quotes extracted from participant transcripts. Example of codes that were identified and developed into themes is presented in **Table 4**. Dropout participants were also invited to interview via email twice, but there was no response.

**Table 4:** Qualitative codes subthemes and TFA domains.

Example codes	Subthemes	Main theme as mapped to TFA domain
Validating UPFA Confidence in group settings Aware UPFA is more than food and underlying unsolved issues	Liberate being acceptable for UPF addicts.	General acceptability
Self-confidence Initial confusion Self-awareness Learning style awareness Valuable support network Happy to receive more information Structure Felt safe and validated Aware not alone or the first in recovery Emotional awareness Validating UPFA Confidence in liberate Self confidence in emotions Confident in discussing UPFA Aware that other are struggling with UPFA Aware of public health crisis of obesity Aware of living older by more unhealthily Confident that UPFA can help Indicate possible barrier Confidence in referral Aware of UPF being mainstream go to for people unaware Aware of the lack of understanding on packaging Aware of lack of education around UPFA Aware of food industry Self-discipline	Liberate participants enjoyed the educational parts of the intervention Liberate participants practiced with new tools to support abstinence or harm reduction in UPFA. Liberate provided valuable peer-to-peer support during the sessions and via WhatsApp. Participants felt validated, and liked having the label of an UPFA	Affective attitude
Awareness of the effort needed, and the tools were there if needed Self-critical Aware of some shame possibly due to stigma around addiction Wanted to find somewhere alone to do the course Self-efficacy Aware of learning style Aware that others might find it too much Feels it’s now or never	Effort required to participate in Liberate Degree of willingness to put UPF recovery first Methods of improving Liberate for future participants.	Burden
Not aware of any ethical concerns Animal welfare ethics Aware of safety and confidentiality Happy with group support	A safe space Animal welfare	Ethicality
Validating UPFA Aware of reasoning behind UPF Aware of lack of education around UPFA Believes many will rely on UPF Believes real food is cheaper Aware that others might find it too much	Understanding of Liberate, UPFA, and the science Greater awareness of tools available	Intervention coherence
Food cost more Aware amount of UPF given to children Change made throughout family Reduction in UPF purchases balances with real food Not affected by socialising	Change in spending patterns Time sacrifice	Opportunity costs
Reduction in “food noise” Validated UPFA Clarifying UPFA vs. real food Aware of addictive behaviour with certain foods Support Structure and support Self-awareness Reduction in “food noise” Self-control Self confidence Aware of addictive behaviour with certain foods Self-efficacy Humility towards change	Supported self-awareness and self-growth Supported improvement in UPFA symptoms, impulsive eating and other outcomes Structure and support as a mechanism for effectiveness	Perceived effectiveness
Self-efficacy Emotional eating Confident in group participation Self-confidence Low self-confidence with tech Learning style awareness Self-awareness Humility towards change Confidence in self-referral	Self-efficacy and self confidence in participating in Liberate from self-referral to attending and completing sessions Engaging in the activities outside of the sessions Hope beyond Liberate and weekly support sessions.	Self-efficacy
Happy to have GP referral Barrier to referral Confidence in referral	GP Involvement, NHS Referral and desire for intervention involvement.	Integration with health services

### General acceptability

All participants’ interviews expressed how “amazing” Liberate had been to their recovery, the ongoing peer-to-peer support, and understanding of neuroscience in UPFA. They considered Liberate to be more than a weight loss intervention, but rather an intervention that has helped them to better understand their condition in a biological and psychological manner. Participants expressed how well the programme aligned with their expectations and needs, viewing it as suitable and supportive for individuals with UPFA.

#### Liberate being acceptable for UPF addicts

“I felt welcomed in amongst people who knew what was going on rather than me sitting there thinking, I don’t get this, I don’t understand what they’re talking about.”

(P1, 139-141)

“It’s hugely important. I think going back to, if I’d have done this right at the beginning of my journey, I think it would have opened so many of my eyes to things that I didn’t even know were going on.”

(P11, 211-213)

“The fact that we’ve got an ongoing weekly session on a Tuesday, it’s like wow, that’s amazing.”

(P13, 240-241)

### Affective attitude

Affective attitude notes participants’ thoughts and feeling towards Liberate. Most participants described the education as liberating, the addiction-focussed tools were described as being helpful, and the peer support was highly valued. However, some participants found some parts uncomfortable and offered suggestions to improve the acceptability of Liberate. Overall, the majority of participants felt validated through participating in the Liberate programme.

#### Liberate participants enjoyed the educational parts of the intervention

The structure and delivery of Liberate was received well by participants. Some participants described the weekly sessions as an opportunity for “me time”. Learning that they were not to blame for past food choices and understanding why it is not their fault were extremely valuable.

“…It was the fact that you had said, “It’s not your fault.” … That really stuck in my brain as like a really memorable moment … That sort of like takes the pressure off you and then you think then, “It’s not my fault, I can do this.”

(P10, 96-101)

“… I was blaming myself a lot for getting into the situation and so when someone said to me, “It wasn’t your fault to get here, but you need to do something about it now,” I think that helped a lot.”

(P3, 158-160)

For others, Liberate allowed participants to better understand UPFA experientially and scientifically allowing for better understanding of their condition and reactions to certain foods.

“And you quite rightly said, it’s no different from a drink or a drug addiction, it’s to be treated the same. And that’s the key thing, I think, that people don’t, they just look at you and think, “Oh, she’s fat, she’s lazy, she’s greedy.” Maybe she is, but maybe there isn’t, sometimes maybe there is something not quite right there. And the mid-brain is like taking over.”

(P10, 116-120)

“I don’t think we spoke about calories on the Liberate course. We just spoke a lot about how your brain works and how your body then processes that.”

(P4, 109-113)

#### Liberate participants practised with new tools to support abstinence or harm reduction in UPFA

Participants mentioned that using the tools daily, via the workbook, helped them keep a focus on strengthening their abstinence rather than a focus on trying to lose weight.

“It took me two weeks to get round to it until I decided that there wasn’t much point going through this programme unless I was going to take out the very thing that I thought was the most obvious … I have changed my diet quite intensively along the lines of the red, amber, green model and will continue to do that. I think I’ll always know if I’m eating something that’s R [red], A [amber] or G [green].

(P9, 102-107)

##### Liberate provided valuable peer-to-peer support during the sessions and via WhatsApp

Participants found the sharing and support part of the session to be valuable to their recovery, especially being able to ask questions and air what was important to them at that point emotionally.

“But also having that contact with the WhatsApp in between. I think that was invaluable, so that we could all kind of debrief and then talk about stuff that we’d learnt about, but then, and because of the people in the group, some have got more experience and more knowledge than others so lots of sharing of additional resources as well.**”**

(P7, 84-87)

WhatsApp was particularly beneficial as it enabled instant connection with other individuals with UPFA at any time if navigating emotional fluctuations.

“I’ve got the luxury of having a brilliant WhatsApp group that are, you know, we’ve gelled so well and we’re, you know, we talk on almost a daily basis, people are putting stuff in there, and it’s so, so supportive, that I don’t feel that, a huge need to have just sort of the sharing check ins, because I’m getting that on a daily, or a couple of daily basis, on my WhatsApp group,”

(P7, 217-220)

However, some people found the WhatsApp groups challenging if other participants were not engaging or triggering when certain foods, recipes, or pictures of food were posted.

“it wasn’t really helpful hearing about other people’s slip-ups”

(P5, 157-158)

#### Participants felt validated and liked having the label of a UPFA

Attending the programme provided participants with a sense of relief as they were better able to identify what they were going through and what the condition actually was. This validation and identification of being addicted to ultra processed food was very important especially in planning the next stages of their healing journey.

“When I managed to hear about Liberate and was sort of like, “Yeah, I’ve got a food addiction,” I didn’t need anyone to tell me. And I thought, “Right, yeah, that’s it, that’s the last piece of the jigsaw.” I filled out all the forms and everything was successful in getting on.”

(P10, 390-393)

“I think that’s probably when I first did it, that was the moment where I was like, “Oh lord, oh god there’s a problem here.” Because it’s not only information for you guys, but it helps the person go, “Wow, okay I’ve got a problem.”

(P3, 482-484)

### Burden

Burden was reflected in the effort required to engage with the course, including workbook activities and personal reflection, yet participants expressed a strong willingness to prioritise their recovery, even to the extent of a sense of urgency to participate. Some of the burden experienced led to participants suggesting improvements.

#### The effort required to participate in Liberate

Participants recognised the effort that it took to participate was outweighed by the benefits gained from their involvement.

“I’ve cooked more in the last six weeks or eight weeks than I have in the previous years you know, so it’s positive that it does make a difference. But it doesn’t come without effort”

(P9, 266-268)

“It is a big thing to take on. But, as I say, totally worth it.”

(P11, 196-197)

Attending weekly sessions was not perceived as burdensome but completing the workbook twice daily was time consuming, where some participants would sometimes forget and felt as though they had failed by missing a day.

“I used to beat myself up by the fact I didn’t complete it every day, you know. I’d find myself three or four days, oh my God, I haven’t done anything, you know. But on the upside it was there when I needed it and I’ve been able to go back to it and look at things. So, that was really good, I enjoyed that, yeah.”

(P1, 161-163)

“… I have a real issue about writing things down, I don’t do any journaling, even from a young age, I’ve never had a diary, every time I write something down, it feels false, it doesn’t feel truthful at all.”

(P6, 341-343)

#### Degree of willingness to put UPFA recovery first

Some participants found that the burden came from their willingness to participate during the sessions; this effort was necessary as prioritising recovery helped them in other areas of their lives, but maintaining this effort was challenging at times.

“You need to be committed to go every week, fair enough, and do a little bit. You can’t just think, “I’ll log in,” and expect it just all to be there in an hour and you’re gone.”

(P4, 184-186)

“No, not really, I fitted it in with my life, you know? I sort of like maybe one night when I went to bed, I thought, “Oh, I’m going to watch one of those videos,” because most of the stuff was like an hour long, which was just right. And then because it was on like YouTube and that you could pause it.”

(P10, 160-163)

Some participants expressed a sense of urgency, that this was their “last chance” to do something, reiterating their decision-making process to put their recovery first.

“So beforehand, really nervous. I think because it really did feel like last chance”

(P8, 92)

“but maybe this hit the point in my life now where I was ready for it-because it had got to a point where, like, if we’re thinking of having kids, I was overweight, pre diabetic, my blood sugar was terrible. That would be so irresponsible of me to try and have kids now. So I did feel like this was my chance, which is why I’ve probably tried so hard with it.”{it}

(P8, 496-500)

#### Methods of improving Liberate for future participants

During the interviews, participants gave suggestions to improve Liberate for future participants. Most participants suggested having access to the learning materials pre and post session, especially so if they wanted to spend more time on specific sessions this would allow them to do so. There were also some suggestions about the order in which sessions were delivered could further improve acceptability.

“I think the lack of getting the slides for me was an obstacle and I understand there might be practical reasons for that.”

(P9, 109-114)

“That’s why I think, when I said to you before about maybe having a bigger gap between each of the teaching points might have been helpful to give more time to just, put it into practice more and feel more confident that you’ve got each thing under your belt before the next bit is introduced.”

(P7, 147-150)

“I’d bring the addiction stuff in first. I would hit them square between the eyes with the addiction stuff.”

(P8, 117-118)

One participant reported struggling with the digital literacy required to access the Liberate workbook.

“When I couldn’t make the interactive book work, it threw me. It didn’t matter what I did, I couldn’t … And so, I was immediately on the back foot. That took me virtually the whole of the sessions to get over.”

(P1, 64-69)

### Ethicality

Some participants felt the content was understandable, relatable, and something they resonated with. The level of privacy and anonymity they had during the sessions created a psychologically safe and non-judgemental space. This contrasted to commercial weight loss groups. Concerns around animal welfare were also mentioned.

#### A safe space

Level of privacy and anonymity was recognised by participants and how this was important for creating a safe space.

“Because it was also a bit anonymised anyway, so I didn’t feel exposed or anything, that’s the thing as well that made me confident about participating.”

(P12, 335-337)

“Because people didn’t have to have their cameras on or put their names on the thing, did they? So, if they wanted to remain anonymous to the rest of the world, rest of the group.”

(P1, 235-236)

Some participants compared their Liberate experience with past interventions in commercial weight loss groups and stated that they felt less judged during Liberate and less pressure to lose weight but rather to focus on the reduction or elimination of the “addictive” foods they deemed to be problematic and a hindrance to their recovery.

“At the Slimming World, Weightwatchers, commercial places, you go, you get weighed and then you sort of like, you’re not shamed, but you feel guilty. And you’ve got to lose every week, you’ve got to.”

(P10, 121-122)

#### Animal welfare

One participant mentioned ethicality concerning animal welfare which showed a conflict between their recovery and their personal values surrounding animal welfare and suggested how Liberate can incorporate awareness around this area.

“the only ethical concern I would … well it’s promoting eating a lot of meat produce, and animal products.

And I’m not vegan or vegetarian, but I am conscious about that.”

(P12, 197-199)

“And just having maybe … giving some advice on where we can get quality produce from animals that are … So maybe having some tips, or more about how we can get nice quality … I think you mentioned a website to see the different farmers markets in cities, you mentioned that. So it’s just tricky in practice to go there on a Saturday, since I work on weekends.”

P12, 204-210)

### Intervention coherence

Intervention coherence demonstrates the extent to which participants understood Liberate and how it works.

#### Understanding of Liberate, UPFA, and the science

Participants reported understanding the intervention and how it intends to help people with UPFA. They were able to demonstrate their understanding by explaining how UPFA affects the brain and how the tools and support are able to help their recovery.

“I think it is because it’s learning why. You weren’t born addicted to processed food. You come to like it. So you need to then realise why you’ve become like it, which is what the course tells you, to then how you can stop yourself being like it and make your life easier going forward by having the coping mechanisms and the strategies in place to know what to do to correct it.”

(P4, 198-202)

“I went back through my notes this morning and looked at, some of the information is just boggling that we don’t know what the food is doing to us. I think it’s hugely significant that we know what the food is doing and how addictive it is.”

(P11, 213-215)

#### Greater awareness of tools available

This shows how well participants understood the intervention and its rationale, including explanations and resource accessibility.

“you had science behind it and people had tested it”

(P10, 116)

“I don’t think we spoke about calories on the Liberate course. We just spoke a lot about how your brain works and how your body then processes that. Much more of a biological reason behind it, and the way the brain functions as opposed to what you put in your mouth. It’s how your body processes that. It’s completely different.”

(P4, 109-113)

“But the information, no, the science stuff was excellent, I really liked that”

(P11, 295)

“you explained how the brain works, you explained about ultra-processed food, all the things it did.”

(P10, 186)

Some participants were happy to receive more information beyond the core curriculum as the course continued:

“I would read scientific paper, and scientific paper, and scientific paper about any of this sort of stuff. I would just consume any information I was given.”

(P8, 452-453)

### Opportunity costs

Opportunity costs demonstrates the degree to which benefits, profits, and values had to be sacrificed in order to commit to Liberate. Participants reported both the initial financial and time investments involved in dietary change but ultimately viewed these as worthwhile trade-offs, especially as cravings diminished and food spending decreased.

#### Change in spending patterns

Some participants mentioned the change in financial cost they perceived the diet had caused upon starting Liberate. Some participants recognised that the initial cost was higher; however, as the recovery process advanced and their food cravings diminished, their food expenditure came down and the realisation of money better spent to provide the family a higher-quality diet also improved.

“I do think it’s a lot more expensive to go whole foods, for sure, I went to the supermarket with my husband a couple of weeks ago and there was no ultra-processed in there and we spent like, I want to say another 20% on what we actually usually spend.”

(P3, 420-426)

“Although I guess that’s a very initial cost, but actually as time’s gone on it has then made me realise that actually I’m feeding my children lots of UPF and actually we need to change as a family.”

(P5, 309-311)

### Time sacrifice

Some participants agreed that the daily tasks set out in Liberate were useful in consolidating their experiences of the intervention. However, some participants found that the daily commitment required to document their experience and complete assigned tasks was perceived as overly time-consuming despite recognising the value in the suggested activities.

“And I really liked that you shared extra books and links to videos, to do that in the week, and get even extra resources, that was really good.”

(P12, 117-119)

“I liked the workbook with the daily, “What have you eaten? What have you planned to eat? What have you eaten?” all of those kind of prompts”

(P11, 129-131)

“It takes time, doesn’t it, to accept that this takes real effort.”

(P11, 265-266)

However, some suggested that having the ability to review sessions or access slides for further reinforcement would have enhanced their confidence:

“I think it would be really useful to have the slides available before the session starts so I can read them and take notes.”

(P9, 314-321)

“I almost want it in a way-like if that was an app and you could add in say cheese or blue cheese, and you could search for it and it would tell you the last days you put it in.”

(P8, 170)

### Perceived effectiveness

Perceived effectiveness denotes the degree to which participants perceive Liberate will successfully fulfil its intended purpose.

#### Supported self-awareness and self-growth

Participants indicated that Liberate facilitated self-reflection on their UPFA experiences across the duration of the intervention and beyond which was a key aim of Liberate.

“If I’d made one round of sandwiches, I’d make two or three and graze. Put them in the fridge and then graze on those the rest of the day. Why did I do that? Because I was addicted to food.”

(P1, 377-380)

“It’s small things like that. It makes you think twice about, well, I don’t need bread in the house because all I would do is make toast to put butter on. So, if I haven’t got the butter, I don’t need the bread.”

(P1, 356-358)

##### Supported reduction in UPFA symptoms, impulsive eating, and other outcomes

Participants reported a reduction in symptoms highlighting the comparison of their symptoms before understanding UPFA versus after attending Liberate.

“Over the program I’ve realised why I have this, what triggered it which was quite a bad situation at work … It [food] was all I ever thought about all day, every day, it was just so much guilt … I mean, if I could put it simply, I think it’s changed my life in a thousand ways, and I can get really emotional about this because I’ve been dieting since I was like seven.”

(P3, 48-58)

Participants also noted a reduction in impulsivity with some reporting an ability to avoid such behaviours without the difficulties they encountered around addictive foods prior to Liberate.

“And my red list is still there. I’m just, “No don’t, step away from the bread.” So yeah, that’s just on the red list. So I haven’t had it for months and months now.”

(P4, 270-272)

Some participants gained enough confidence to be able to manage their food recovery in social situations without their eating becoming emotional and impulsive.

“It’s not an emotional thing that much anymore, like I look at biscuits and be like, “It’d be lovely but no thank you, I’m okay.”“

(P3, 494-496)

#### Structure and support as a mechanism for effectiveness

Participants expressed the importance of the ongoing support found in the weekly support sessions and how group dynamics played an important role in their continuing recovery

“we were sharing, we had time to build up that trust, to make ourselves vulnerable and share, whereas in the wider group, also there’s that sense of who’s going to be in there this week?”

(P7, 225-228)

“so to have that external sense of, and I know you the weekly sessions will shift and change but I suppose it’s that sense of being part of a bigger community and that community’s there.”

(P2, 448-450)

### Self-efficacy

Participants described an initial apprehension at the start of Liberate, but gained confidence through participation, including using the tools beyond the course, but needing to engage with ongoing support.

#### Self-efficacy and self-confidence in participating in Liberate (from self-referral to attending and completing sessions)

Whilst many had attended commercial weight loss programmes, confidence to even self-refer to Liberate for many participants was significant as it meant acknowledging their addiction to UPFs. This also meant they needed to feel ready to deal with shame and stigma associated with the term “addiction”. “When I signed up, I was a bit anxious about what I was signing up for because it’s an unknown thing, isn’t it?… I have to actually admit to other people that I’ve got a bit of a problem with food, which I didn’t realise.”

(P4, 34-38)

Confidence further improved as participants engaged with the programme both during sessions and outside of the sessions through homework and activities in their workbooks. “I think having that sense, a support network handy now, it improves my confidence compared to where I was before. I know that I can do it, because I have done it.”

(P7, 292-297)

##### Engaging in the activities outside of the sessions

Participants reported increased confidence in using the tools during the 6-week intervention particularly the workbook.

“I think having that sense, a support network handy now, it improves my confidence compared to where I was before. I know that I can do it, because I have done it, so, for long periods, so yeah I think I just need to, having been derailed, have a sustained period where I’m back on it, to keep going, and I think that’s been the problem, is feeling that I didn’t have the support around me to help me sustain it for a reasonably period.”

(P7, 292-297)

However, some suggested that having the ability to review sessions or access slides for further reinforcement would have enhanced their confidence.

“I think the lack of getting the slides for me was an obstacle and I understand there might be practical reasons for that.”

(P9, 109-114)

One participant mentioned that they struggled with the digital literacy side of accessing Liberate workbook.

“When I couldn’t make the interactive book work, it threw me. It didn’t matter what I did, I couldn’t … And so, I was immediately on the back foot. That took me virtually the whole of the sessions to get over.”

(P1, 64-69)

#### Hope beyond Liberate and weekly support sessions

Many participants discussed if they continued using the tools they had been given through the programme, they were confident they could maintain progress made illustrating hope.

“I’ll maintain it, I’m sure about it because I just don’t feel the need for it [UPF] anymore.”

(P3, 494)

“And I guess if I actually sit and do the work and do the workbook and come to the group sessions, then I feel quite confident. That makes me feel confident that I will carry things on and that I will maintain yeah.”

(P5, 395-398)

However, some participants did not yet feel confident in using the tools on their own and were grateful that the weekly support group while still building confidence.

“If this was goodbye, and we didn’t have the Tuesday thing. I think I’d feel a bit like, ooh, cut loose a bit. So having that support every Tuesday, it’s priceless, that’s priceless.”

(P13, 256-259)

### Integration with health services

#### GP involvement, NHS referral, and desire for intervention involvement

This theme reflects participants’ desires for primary care and other formal health services to acknowledge and address food addiction and bring interventions like Liberate into mainstream healthcare.

“I went to the doctors for help and didn’t really get much help really, he just said, “Well, go on a diet and walk more.”

(P10, 383-384)

“The more we bang on the GPs doors, the more they’re going to start listening because you were just, let’s be honest, food addiction was, “Oh, you’re just greedy. You’ve got “no control.”.”

(P10, 107-111)

Participants stated that GP involvement could provide support and reductions in long-term health costs but often felt GPs did not understand UPFA.

“I would be absolutely delighted that my GP had suggested something. I would probably be a bit offended at first, but … I would be really pleased, especially if I had health issues connected with it. Yeah, I’d be pleased.”

(P11, 179-182)

“If the NHS would sort of like take it on board and roll it out, they’re just looking at this little picture. I mean, it might cost some money to do it, but the money they’ll save,”

(P10, 414-416)

## Discussion

This study aimed to explore the feasibility and acceptability of an intervention aimed at reducing symptoms of ultra processed food addiction (UPFA). A total of 86 participants were recruited into the feasibility study, and 15 participants dropped out at the end of the intervention and a further 12 participants dropped out at follow-up, demonstrating a 69% retention rate. This suggests that this intervention was feasible to recruit to and can retain participants suggesting the acceptability of study procedures and design. Qualitative data identified overall acceptability of the Liberate intervention, with some improvements for refinement.

Quantitative findings of secondary outcomes showed significant improvement in psychological measures, reductions in food addiction as measured by YFAS and CRAVED and increases in mental wellbeing as measured by WEMWBS. In using WEMWBS, a clinical significance threshold in a community sample is seeing a 3-point increase, whereas this study showed a 6-point increase (43).

Whilst weight loss was not the goal of this intervention, participants did report a statistically significant difference in weight; however, this was not clinically significant. Collectively these findings suggest that Liberate is a feasible and acceptable intervention, and a more definitive trial should be conducted to determine the efficacy of the intervention at a larger scale. The quantitative results correspond with other studies already published that also show that education peer support and an abstinent approach to UPFA treatment can have both physical and psychological benefits (3, 13, 21, 46, 47).

The results showed that it was feasible to conduct Liberate via online methods using a coach-led model over delivering 8 sessions over a 6-week period. Only a small number of people struggled with the technology, and in a future iteration, more support should be provided to ensure participants are supported.

These findings are consistent with previous studies of similar programmes (13, 48). Over the course of the intervention, participants reported a greater sense of control of cravings and eating behaviours, and a reduction in cravings and a greater sense of wellbeing—this is supported by the quantitative data (particularly the YFAS and WEMWBS data). Participants highlighted the importance of structured abstinence from UPF, which further reinforces the need for a new approach in the treatment of eating disorder services only working with moderation. If abstinence is the way patients want to go forward, they should be supported, and so research into priority areas for this particular population should be explored further especially as this aligns with shared decision making and patient empowerment of their own health and well-being. Many also mentioned how important it was to their recovery to have peer support and accountability and a platform for this to be available. These data reinforce the necessity of a multifaceted approach to treating UPFA and as a substance use disorder (SUD), by combining behavioural strategies with education and social support mechanisms.

A key strength to this study is its mixed-methods approach which demonstrated preliminary statistical impact of the intervention and acceptability from the participants’ perspective. This enabled the study to offer numerical evidence and participants’ voices. This study further highlights patients’ acceptability and improvement when accessing much needed behavioural and psychological support alongside dietary changes. This should be highly considered when creating UPFA interventions and particularly leaning on already successful substance use disorder (SUD) interventions. In the analysis, we used the intention-to-treat approach with baseline observation carried forward to replace for missing values. This assumed that there was no improvement in outcomes in people who dropped and is a conservative approach from that point of view. However, this could also be considered a limitation as we cannot rule out any deterioration during this period of time.

There are other limitations to consider too. For instance, sample size, while sufficient for feasibility testing, is not powered and limits generalisability. Additionally, the study relied on self-reported measures which can introduce bias due to accidental or deliberate under- or over-reporting. The methodology did not include a control group, nor were we able to collect data from or interview those that dropped out. It would be useful for future research to use objective measures such as healthcare professional-led weight and waist circumference measurements, or dietary tracking to validate self-reported outcomes (although tracking may be triggering for this population group). Additionally, the programme was delivered free of charge, and it is unclear how introducing a cost would affect uptake, retention, or outcomes. Future studies should explore cost-effectiveness and sustainability in real-world settings.

This piece of research is a novel contribution to the field of UPFA and demonstrates that a UPFA-based intervention can be feasibly implemented online for individuals struggling with UPFA. There are parallels between substance addiction and addictive eating, but there are not many studies that have tested addiction structured treatment from addiction sciences for UPFA. These findings begin to provide evidence for using a structured programme for supporting people with UPFA but require confirmation through future more definitive trials.

To explore the long-term efficacy of Liberate, more people and longer follow-up times would be needed—ideally a randomised controlled trial to establish a causal effect of Liberate on the outcomes. Additionally, further qualitative research could help us understand the psychological characteristics underlying recovery from UPFA such as changes in positive outlook, self-identity, emotional regulation, and social connections as this could help us refine future interventions.

Since the results so far have been promising, it would be worth considering adaptation of Liberate to different cultures and testing for acceptability. We could also consider different modes of delivery such as digital platforms with pre-recorded audio-visual content ensuring that peer support is still available to participants. This would provide an economy of scale, the cost-effectiveness of which could determine whether UPFA interventions should be widely implemented as a public health measure. Lastly, consider prevention rather than cure, and educating new families and young people about the effects of UPFA on brain development.

## Conclusion

This study shows us the potential feasibility and acceptability of UPFA-based support treatment towards long-term recovery. This study adds new insights into addiction-based interventions aimed specifically towards people experiencing UPFA. This research lays the foundations of further research where the limitations can be addressed to further look into better treatment of UPFA.

## Data Availability

The raw data supporting the conclusions of this article will be made available by the authors, without undue reservation.

## Appendix

Quantitative appendix

Time points 1 = baseline; 2 = post-interventions; 3 = 6 month follow-up

Time points 1 = baseline; 2 = post-interventions; 3 = 6 month follow-up

Time points 1 = baseline; 2 = post-interventions; 3 = 6 month follow-up

Time points 1 = baseline; 2 = post-interventions; 3 = 6 month follow-up

Time points 1 = baseline; 2 = post-interventions; 3 = 6 month follow-up
