# Supplementary material for: A feasibility and acceptability study of liberate: an online, peer-supported, psychoeducational intervention for ultra processed food addiction
Source: Front Psychiatry. 2025 Oct 29;16:1620372. doi: 10.3389/fpsyt.2025.1620372 (PMC12605181; doi:10.3389/fpsyt.2025.1620372)
Supplement: Supplementary file 1 [file DataSheet1.pdf]

TIDieR Checklist

Item	TIDieR Description
1. Brief Name	Liberate: An online psychoeducational programme for Ultra-Processed Food Addiction (UPFA)
2. Why	Individuals who experience ultra-processed foods (UPFs) as addictive may require different support from standard moderation-based approaches. Liberate draws on evidence from Substance Use Disorder (SUD) treatment where abstinence is often the gold standard, while also incorporating harm reduction principles for those not ready for abstinence. The programme aims to reduce UPFA symptoms (measured by YFAS 2.0), lower CRAVED scores, and improve mental wellbeing (measured by WEMWBS).<br><br>Research by Meule et al. (2019) indicates that 15–20% of people self-identify as experiencing symptoms consistent with UPFA, highlighting a population potentially underserved by existing dietary interventions (1).<br><br>Currently, there is no dedicated intervention in the UK addressing problematic eating from a substance use disorder framework. Given rising obesity rates and related comorbidities (2), offering an addiction-focused psychoeducational intervention for UPFA may be a valuable public health response.<br><br>The dietary intervention of Liberate is encouraged but optional. We suggest whole foods and low

Item	TIDieR Description
	carbohydrate, but the main focus is establishing a comfortable pace into abstinence.
3. What (Materials)	Participants require access to a device such as a computer or smartphone, ideally with a larger screen for slide visibility. Materials include an electronic Liberate workbook (completed daily), weekly slide decks presented by the coach, and links to supplementary videos. A temporary WhatsApp group is offered for optional peer support, moderated by the coach to maintain topic focus. Facilitators are provided with a standardised manual to ensure fidelity across groups.
4. What (Procedures)	Each coach-led session begins with group emotional and physical check-ins, providing an opportunity for real-time assessment of participants' emotional states, as well as reflection on the previous week's tasks. Sessions conclude with three minutes of silence for grounding. Daily homework includes gratitude, affirmations, and intention-setting. Content progresses weekly through educational and support-based sessions covering food planning, neuroscience of addiction, hormonal influences, and behaviour change strategies. Coaches deliver content, monitor attendance, email summaries, and prompt group interaction between sessions.
5. Who Provided	Trained coaches with lived experience in recovery from UPFA deliver the sessions. Each coach must have shadowed at least two previous Liberate cohorts before facilitating.

Item	TIDieR Description
6. How	Delivered entirely online via Microsoft Teams in a group-based format.
7. Where	The intervention is accessed remotely. Participants are advised to join from a quiet, private space. All sessions are hosted on Microsoft Teams.
8. When and How Much	Originally run over 6 weeks (8 sessions in total). Weeks 1 and 2 included two 90-minute (+/- 10 mins) sessions per week. Weeks 3–6 included one session per week.
9. Tailoring	Adaptations are made for those with physical needs or dietary restrictions (e.g., vegetarian/vegan versions of the food plan). An additional support group is available for vegan/vegetarian participants.
10. Modifications	Modifications considered for the next iteration were changing from 8 sessions over 6 weeks (with optional additional sessions) to a consistent 8 sessions over 8-week format. Additional support was also developed for vegan and vegetarian participants. No changes were made to the core content.
11. How Well (Planned Fidelity)	No formal intervention fidelity assessment was planned at this stage. A facilitator manual was developed to guide consistent delivery.
12. How Well (Actual Fidelity)	All sessions were delivered as planned on schedule, and within the expected time window (90 minutes ± 10 minutes). Coaches adhered to the programme structure and delivered all core content.

Intervention details with behaviour change terms from behaviour change techniques Taxonomy (3)

Behaviour Change Table Week	Session	Content	Purpose	Behaviour Change techniques used throughout the course
	Beginning of each session and post intervention support sessions	Begins with an introduction of how the previous week has gone and a discussion on how the tasks have gone and what they have brought up for them.	To ease them into using peer support and an opportunity for self-reflection. An opportunity for other members to highlight methods of support for the person sharing.	Social support (unspecified)<br><br>Social support (practical)<br><br>Social support (emotional)<br><br>Review behaviour goal<br><br>Review outcome goals<br><br>Information about others' approval (relation to sharing)

				Verbal persuasion about capability
	End of each session	Sessions end with a three-minute silence where the participant can choose to sit in silence, move about, write doodle whatever feels right to them.	An opportunity for the participant to get comfortable in just being	
Homework	Daily	Gratitudes, affirmations, and intentions	The positive repetition brings their focus to the PFC and less emphasis on the addiction based in the midbrain	Commitment<br><br>Self-monitoring of behaviour<br><br>Information about antecedents<br><br>Monitoring of emotional consequences<br><br>Prompts/cues<br><br>Behavioural practice/rehearsal

				Behaviour substitution<br><br>Habit reversal<br><br>Graded task<br><br>Pros and cons
Week 1	Session 1<br>Education	Introduction to the food plan, essential macro nutrients	To educate and inform of their food plan	Goal Setting (outcome)<br><br>Problem Solving<br><br>Action planning<br><br>Review Behaviour goals<br><br>Information about health consequences<br><br>Information about emotional consequences

				Restructure the physical environment<br><br>Restructure the social environment<br><br>Avoidance/reducing exposure to cues for the behaviour<br><br>Distraction
	Session 2<br>Support	Additional session in week 1 for extra support during withdrawal period Visualisation on being addiction free	To introduce them to visualisation techniques	Information about emotional consequences<br><br>Comparative imagining of future outcomes<br><br>Identity associated with changed behaviour<br><br>Imaginary reward

Week 2	Session 3<br>Education	Introduction to insulin, cortisol and adrenaline	To educate and inform of the purpose of certain hormones and neurotransmitters in the role of addiction	Re-attribution<br><br>Information about health consequences<br><br>Information about emotional consequences
	Session 4<br>Support	Additional session in 2 for extra support during the withdrawal period	To provide additional support for the withdrawal period	Information about health consequences<br><br>Information about emotional consequences
Week 3	Session 5<br>Education	The process of making a something addictive and discussion on ultra-processed foods	To educate on the science of hyperpalitability	Information about health consequences<br><br>Salience of consequences

				Information about emotional consequences
Week 4	Session 6<br>Education	Understanding addiction as a disease to remove self-blame and encourage self-compassion and understanding the causes of addiction; exposure therapy	To educate group on how addiction evolves in the brain and how to continue recovery with special focus on self-compassion. To start exposure therapy	Information about health consequences<br><br>Information about social and environmental consequences<br><br>Information about emotional consequences<br><br>Exposure<br><br>Framing/Reframing
Week 5	Session 7<br>Education	Dopamine and dopamine receptor disruption; dopamine set point theory and dual addiction and feelings wheel; food choices	To educate and prepare for possible dual/poly addiction and explain why this may occur	Information about health consequences

		recovery protection plan whilst out and about; exposure therapy		Information about emotional consequences<br><br>Behavioural practice/rehearsal<br><br>Behaviour substitution<br><br>Habit reversal<br><br>Conserve mental resources<br><br>Mental rehearsal of successful performance
Week 6	Session 8 Education	Importance of connection and support	To explain the importance of support and connection when dealing with addiction	Information about health consequences<br><br>Information about emotional consequences

1. Meule A, Gearhardt AN. Ten Years of the Yale Food Addiction Scale: a Review of Version 2.0. Curr Addict Rep [Internet]. 2019 Sep [cited 2021 Nov 17];6(3):218–28. Available from: http://link.springer.com/10.1007/s40429-019-00261-3

2. World Health Organisation [WHO]. Obesity and Overweight:Fact Sheet. 2023.

3. Michie S, Richardson M, Johnston M, Abraham C, Francis J, Hardeman W, et al. The Behavior Change Technique Taxonomy (v1) of 93 Hierarchically Clustered Techniques: Building an International Consensus for the Reporting of Behavior Change Interventions. ann behav med [Internet]. 2013 Aug [cited 2025 Apr 10];46(1):81–95. Available from: https://academic.oup.com/abm/article/46/1/81/4563254
